# Supplementary figures and images for: Unexplored viral diversity in Siberian cranes and wild geese: metagenomic insights from a global wintering haven
Source: mSystems. 2025 Aug 28;10(9):e00756-25. doi: 10.1128/msystems.00756-25 (PMC12456024; doi:10.1128/msystems.00756-25)

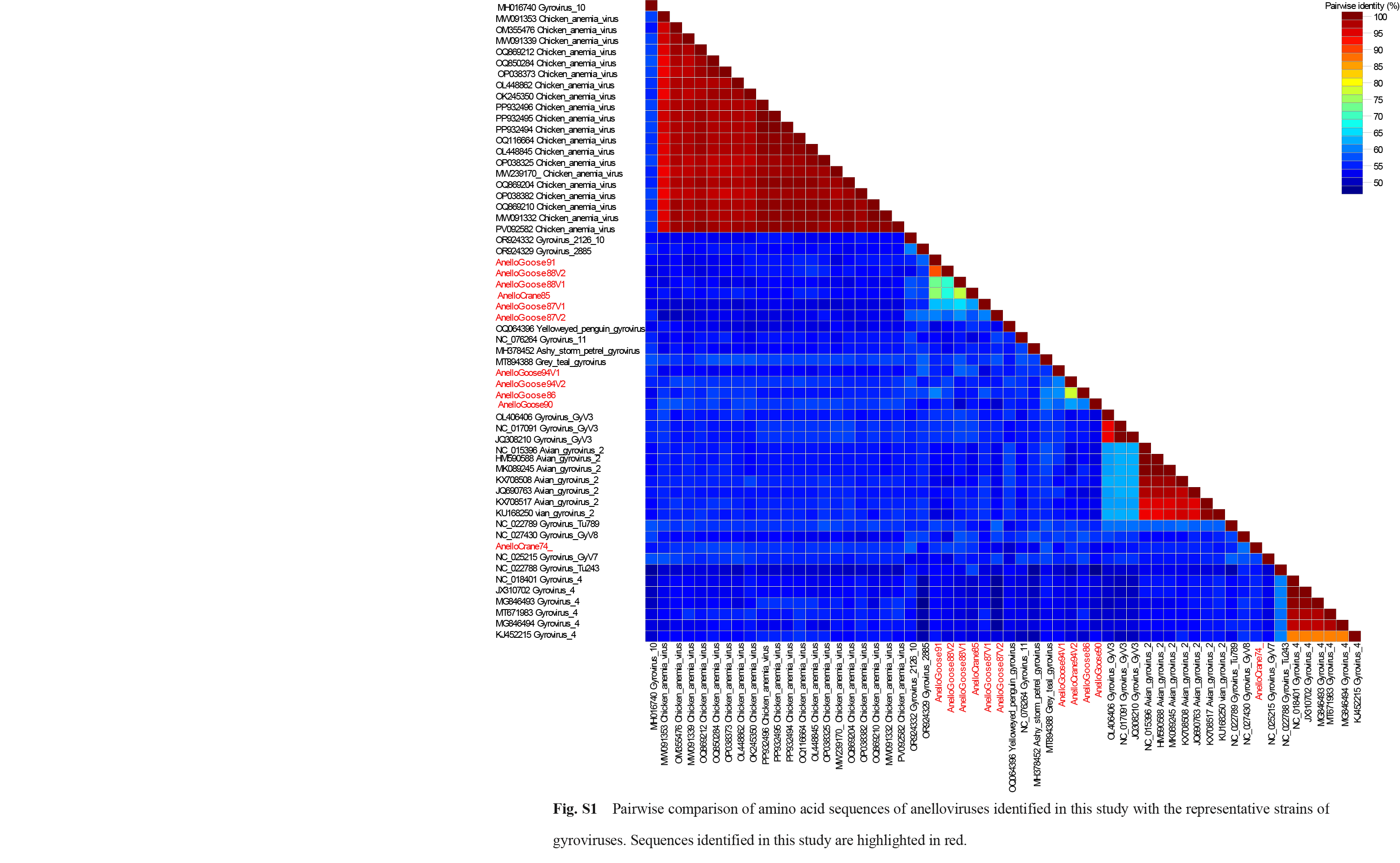

Supplement: FIG. S1 — Pairwise comparison of amino acid sequences of anelloviruses identified in this study with the representative strains of gyroviruses. [file msystems.00756-25-s0001.tif]

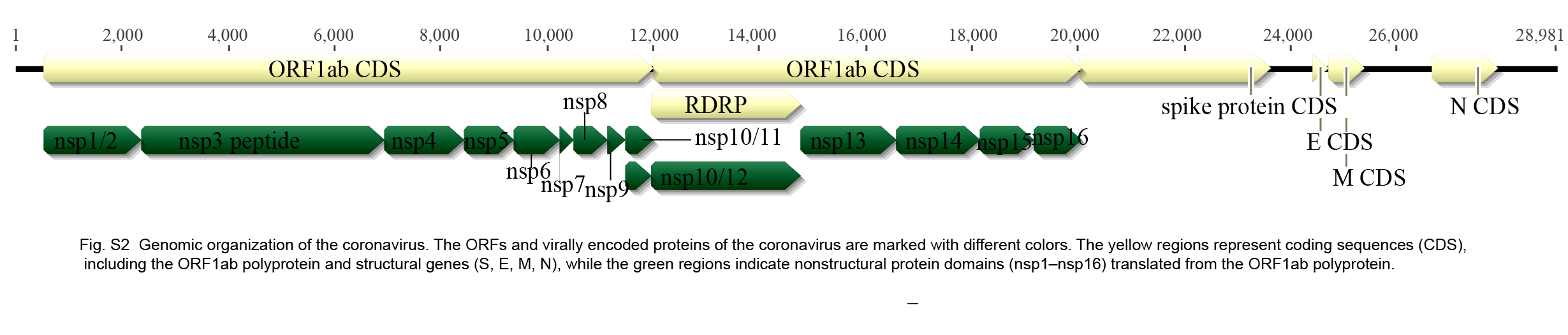

Supplement: FIG. S2 — Genomic organization of the coronavirus. [file msystems.00756-25-s0002.tif]

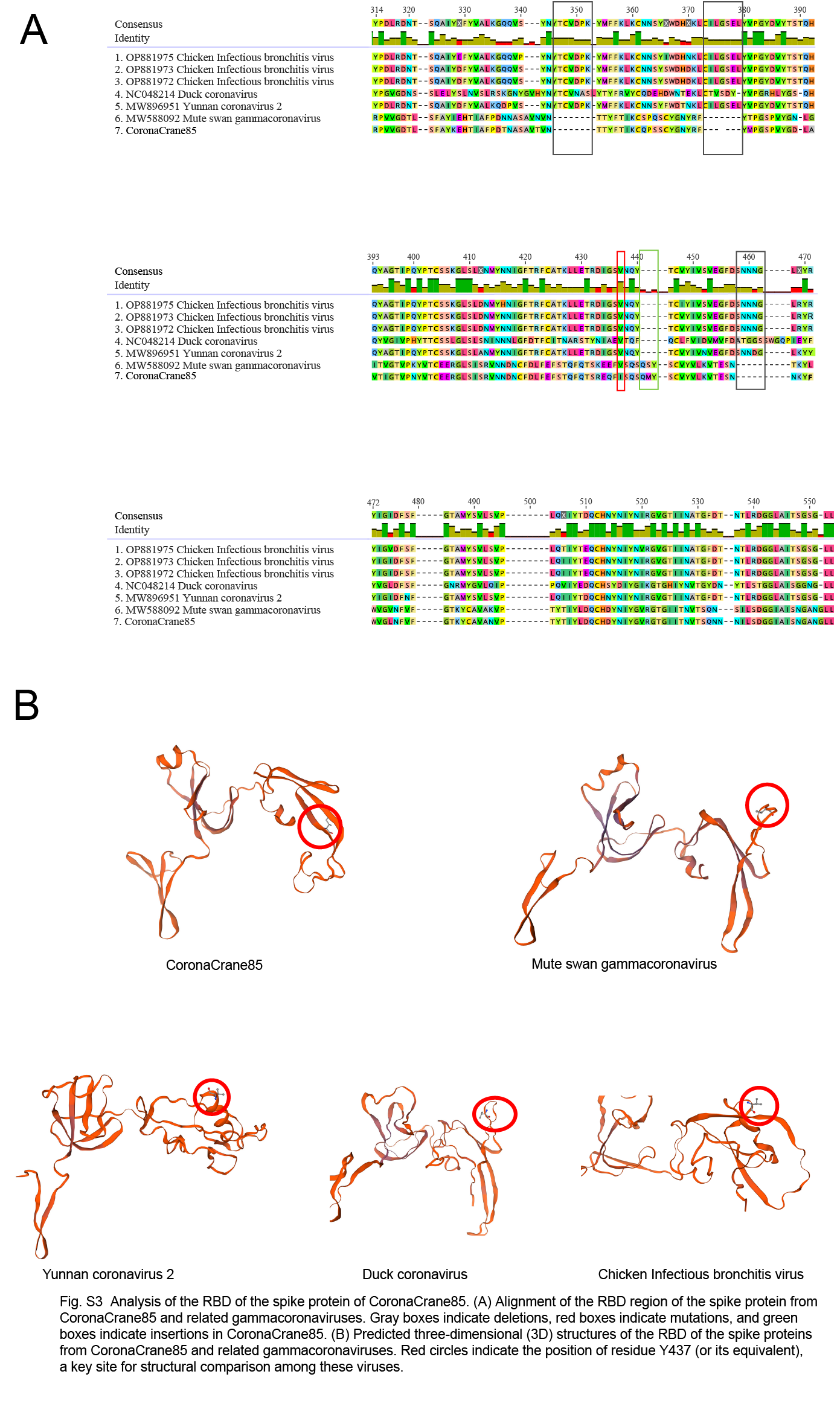

Supplement: FIG. S3 — Analysis of the RBD of the spike protein of CoronaCrane85. [file msystems.00756-25-s0003.tif]

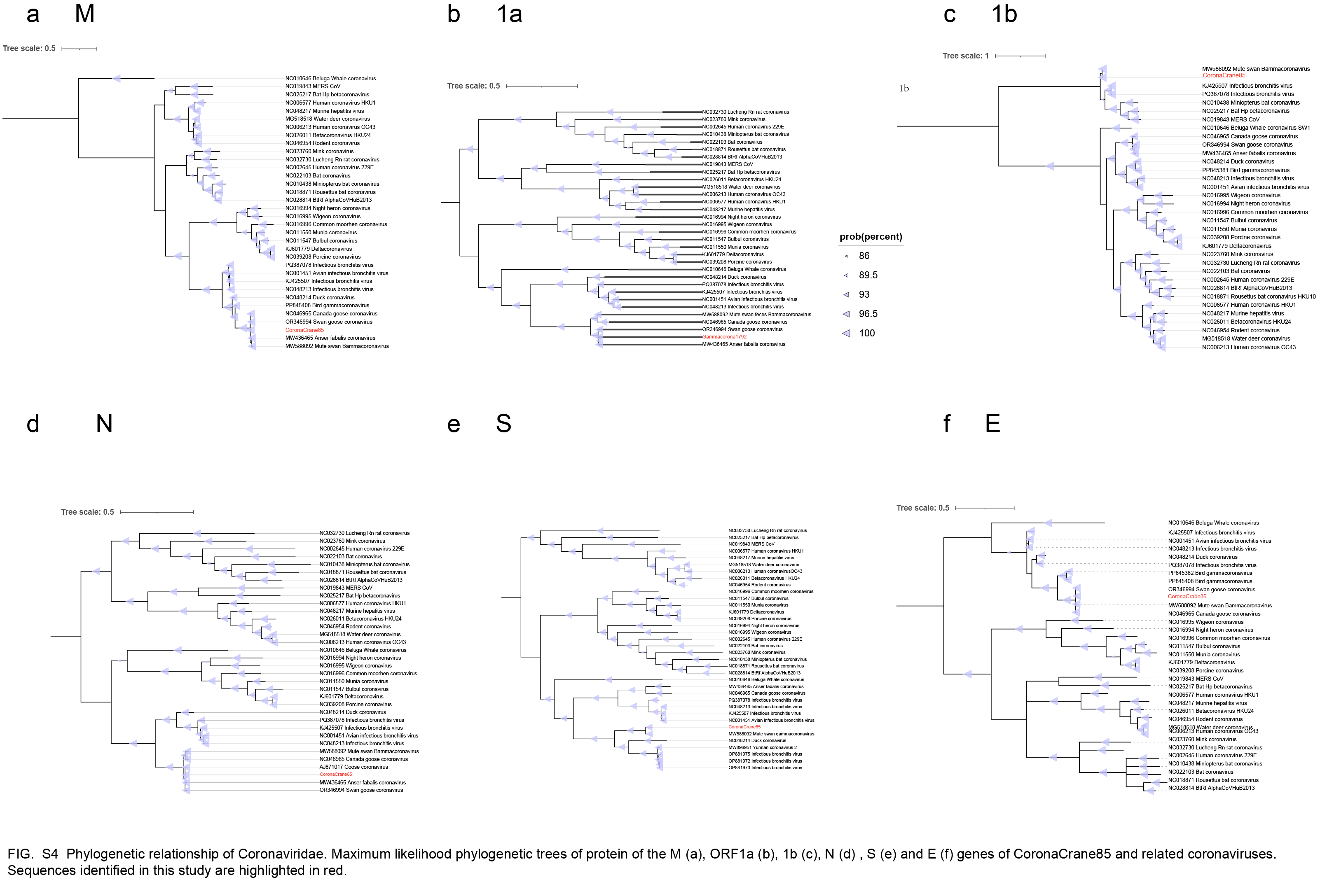

Supplement: FIG. S4 — Phylogenetic relationship of Coronaviridae. [file msystems.00756-25-s0004.tif]

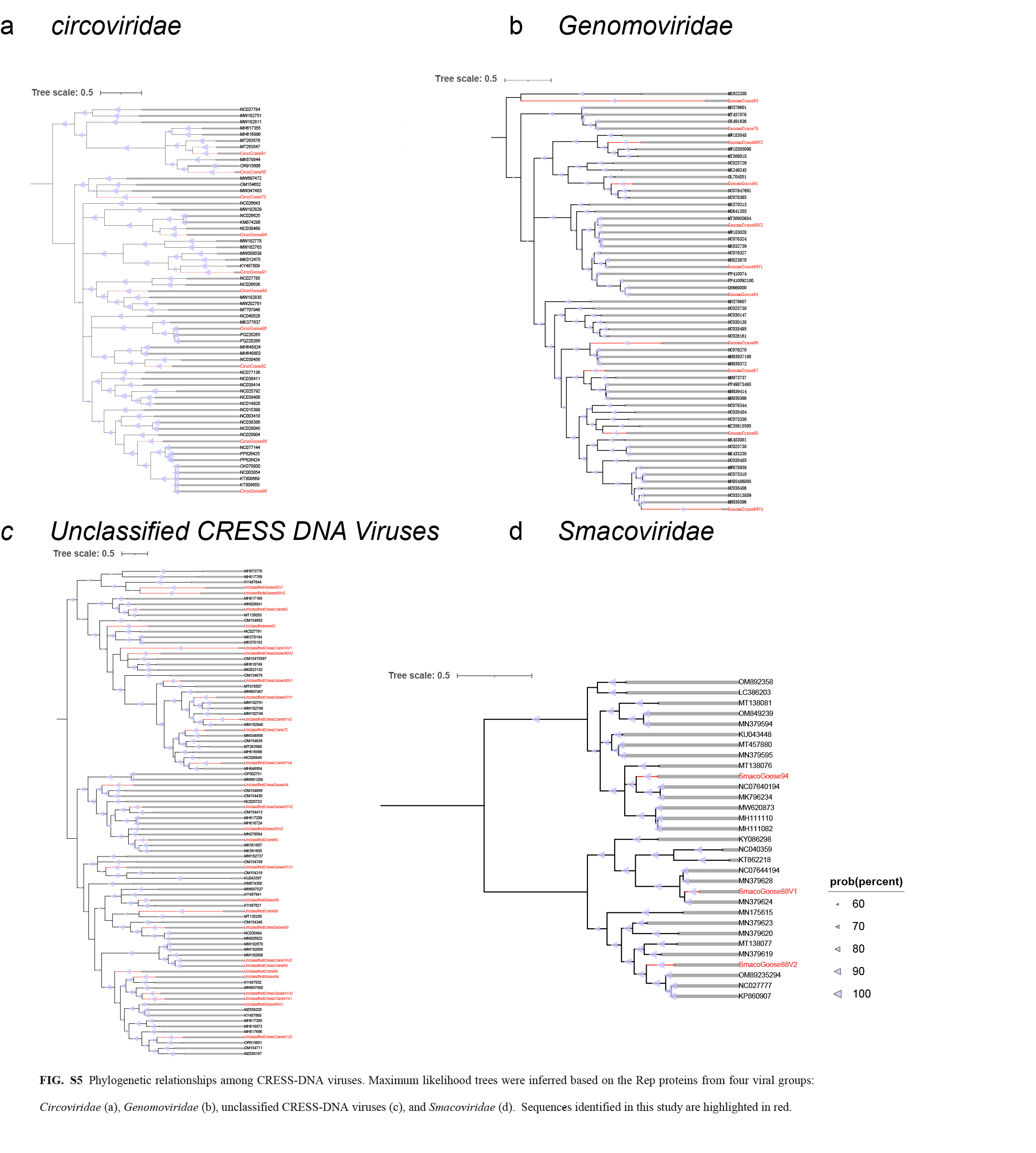

Supplement: FIG. S5 — Phylogenetic relationships among CRESS-DNA viruses. [file msystems.00756-25-s0005.tif]
